# Hydrocyanite of Iron in Epilepsy and Chorea

**Published:** 1829-03

**Authors:** 


18. *Hydrocyanite of Iron, in Epilepsy and Chorea.*—In the transactions of the Royal Academy of Medicine, of Bordeaux, for 1827–8, several cases have been published, illustrative of the efficacy, in epilepsy and chorea, of the hydrocyanite of iron. The medicine was used under considerable diversity of age, temperament, duration of the disease, and state of the arterial system. The dose was half a grain at the beginning, in the form of a pill, gradually increased up to twelve grains a day. We are indebted to the Journal of Foreign Medicine for an abstract of these cases.

